# Effect of 4DryField^®^ PH, a Novel Adhesion Barrier, on Recurrence of Intestinal Adhesions after Extensive Visceral Adhesiolysis

**DOI:** 10.1155/2018/9628742

**Published:** 2018-03-11

**Authors:** Gerhard Blumhardt, Matthias Haas, Stefanie Polte

**Affiliations:** Department of General and Visceral Surgery, Evangelisches Krankenhaus Unna, Holbeinstr. 10, 59423 Unna, Germany

## Abstract

**Background:**

Adhesions occur after up to 97% of abdominal interventions causing chronic pain, infertility, and intestinal obstruction. Various concepts to prevent adhesions have been presented but mostly either have low efficacy or are not applicable in resective intestinal surgery or incomplete hemostasis. In this retrospective one-center clinical trial, the course of patients with extensive abdominal adhesiolysis and application of a recent starch-based formulation, 4DryField PH (4DF), is analyzed.

**Case Report:**

Five female patients (age 65–83 years) underwent extensive open adhesiolysis with application of 4DF gel for adhesion prevention, premixed extracorporeally with saline or Ringer's solution (60–70 mL per 5 g 4DF) for homogeneous gel distribution on intestinal loops and in the peritoneal cavity. In addition, dry 4DF powder was dispersed on the greater omentum and subsequently transformed into a gel by dripping with saline or Ringer's solution directly before abdominal closure. Patients were followed up for more than two years, except for one patient who died after nine months due to metastases. One patient with complex situation due to Gore-Tex mesh in the lower abdomen showed no adhesions at scheduled second-look operation but after six months had relaparotomy for adhesiolysis. All other patients have remained free of adhesions or adhesion-related symptoms during follow-up.

**Conclusion:**

Considering the extent and complexity of adhesions, treatment with 4DF gel for adhesion prevention after open adhesiolysis appears promising. Prospective randomized trials should further elaborate on this clinical concept.

## 1. Introduction

Adhesions are abnormal fibrous structures in the abdominal cavity, mainly encountering after surgery [1]. They occur in up to 97% of patients after abdominal interventions [2–4] and may cause chronic abdominal and pelvic pain, secondary female infertility, intestinal obstruction, reoperations with associated complications, as well as enormous costs for the health-care system [5–8]. Intestinal obstruction is a particularly severe complication with a mortality rate of up to 15% following adhesive obstruction [9]. After surgical adhesiolysis, the recurrence rate may be as high as 55–100%, with a mean incidence of 85% [10]. This recurrence rate is independent of the type of the initial adhesions and will occur regardless of whether the adhesiolysis was performed laparoscopically or by open surgery [11]. Furthermore, recurrent adhesions can be even more extensive than in the primary situation, necessitating further operations [5, 10, 12]. The financial burden of adhesions is significant. Associated costs for adhesiolysis in the United States alone were 1.3 billion USD in 1994 [13]. In Sweden, the annual costs related to small bowel obstruction were estimated at 40–60 million Euros [14]. Thus, adhesion prevention is not only a major concern for patients' well being but also has an enormous impact on health-care costs. However, the efficacy of various agents for prevention of adhesions is discussed controversially [15]. In addition, most devices are limited in situations with incomplete hemostasis or bowel resection and consecutive anastomosis [12, 16–18].

4DryField PH (4DF), a starch-based formulation, was initially developed for hemostasis in cardiac surgery and, in addition, showed strong adhesion prevention capabilities. The substance is approved for hemostasis and treatment of adhesions after surgical adhesiolysis. Mixed with saline solution, this polysaccharide has been reported to be highly efficient in adhesion prophylaxis in experimental and gynecological studies [19–26]. The purpose of our analysis was to find out if these promising results might be transferred to visceral surgery.

## 2. Case Presentation

This retrospective study analyzes five patients with extensive adhesions involving large areas of the intestine and the parietal peritoneum. Five female patients (age 65–83 years, operated between August 2014 and January 2015) presented with severe symptomatic intestinal adhesions following previous abdominal procedures. Three of them were elective, two were acute presentations. All patients underwent extensive open adhesiolysis and were treated with 4DF gel before closure of the abdomen to prevent recurrence of adhesions. To ensure homogeneous distribution of the barrier gel, 4DF powder and saline or Ringer's solution were premixed extracorporeally by adding 60–70 mL saline or Ringer's solution to every 5 g 4DF powder. The powder was first poured into a kidney dish. After adding saline or Ringer's solution, both components were thoroughly mixed until a smooth gel had formed. As the gel remained stable for hours, it could be prepared well in advance of the application. The gel was distributed on the entire intestine and in the peritoneal cavity either via a bladder syringe or poured directly from the kidney dish. In addition, dry 4DF powder was dispersed on the greater omentum and subsequently transformed into a gel by dripping with saline or Ringer's solution, followed by closure of the abdomen. The present analysis is based on a series of complete clinical records, including records from another institution, where one patient had to be treated before being readmitted to our hospital. Patients were followed up for more than two years, except for one patient who died due to metastasizing rectal carcinoma.

### 2.1. Patient 1

**Figure 1:**
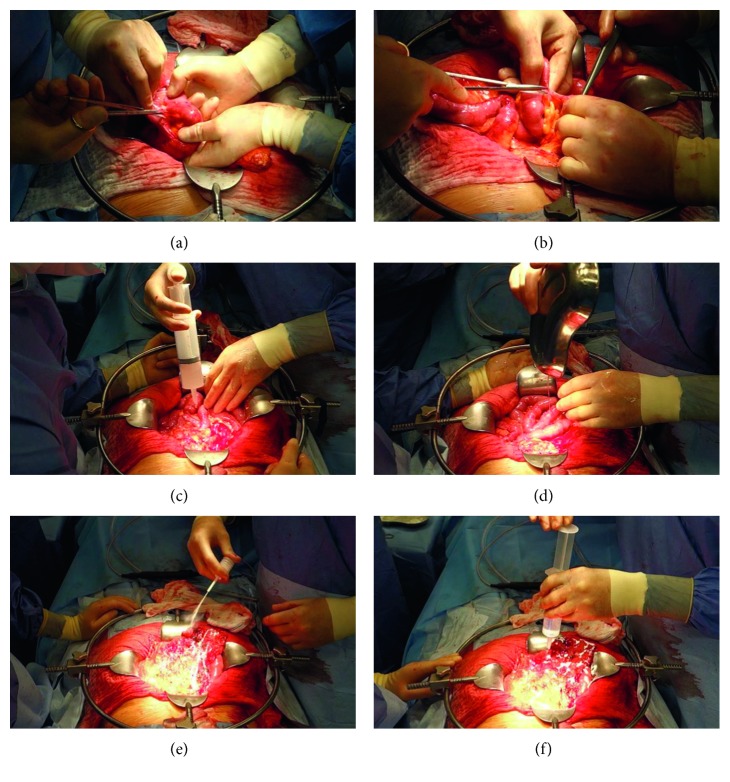
Primary surgery with adhesiolysis and subsequent application of 4DryField PH (4DF) in the first elective patient. (a, b) Dissection of extensive intestinal adhesions. (c, d) Application of extracorporeally premixed 4DF gel via syringe (c) and directly from the kidney dish used for premixing the polysaccharide powder with saline solution (d). (e) Application of 4DF powder onto the greater omentum. (f) Transformation of the powder into a gel by drizzling saline solution onto the powder.

The first patient was 80 years old and presented with severe chronic abdominal pain massively affecting her quality of life. The intake of solid foods was massively impaired. During a history of 14 years, she had been operated twice for abdominal adhesions, at the second intervention with application of 4% icodextrin solution for adhesion prevention. In addition, the patient was hospitalized for conservative treatment of subileus. On the occasion of the present laparotomy, two conglomerates of the small intestine were found (Figures 1(a) and 1(b)). Adhesions were dissected in an extensive procedure in which serosal injury could be avoided. After thorough rinsing of the abdomen, 15 g 4DF premixed as a gel with 180 mL 0.9% saline solution was applied onto the entire small intestine and the peritoneal cavity via a bladder syringe (Figure 1(c)) and directly from the kidney dish used for premixing (Figure 1(d)). Additionally, 5 g 4DF powder was applied on the greater omentum (Figure 1(e)) and moistened with 0.9% saline solution (Figure 1(f)) before closure. In the early postoperative course, the patient developed pneumonia, which improved rapidly under therapy with intravenous antibiotics. She was discharged after 11 days and has remained free of symptoms for 2 years and 4 months.

### 2.2. Patient 2

The second elective patient was 83 years old and presented with an incisional hernia and chronic symptoms of intestinal obstruction ten years after transperitoneal resection of rectum adenoma. At the present laparotomy, the small intestine presented as a tight conglomerate that was firmly fixed to the pelvis. During an extensive procedure, a complete adhesiolysis of the small intestine was performed. Intestinal injury did not occur. After rinsing and meticulous hemostasis, treatment with 15 g 4DF plus 210 mL Ringer's solution, premixed as a gel, was performed. The gel was applied onto the intestinal loops and the peritoneal cavity, and the hernia was closed with a direct suture. The abdomen was closed without insertion of drains. The postoperative course was uneventful; the patient was discharged after 9 days. She has remained free of symptoms for 2 years and 2 months.

### 2.3. Patient 3

**Figure 2:**
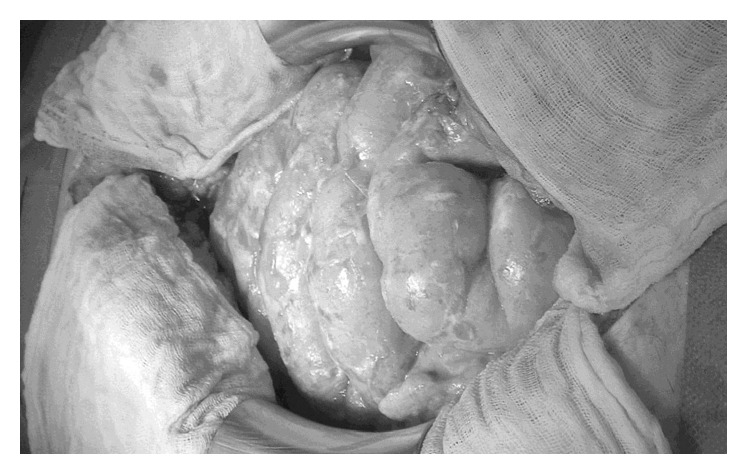
Second-look surgery five days after the primary surgery of the third patient with no indication of new adhesions.

The third elective patient was 77 years old and presented with a massive exacerbation of chronic lower abdominal pain, which did not respond to conservative treatment. Diverticulitis and localized peritonitis were suspected. Previously, she had been operated for ablatio mammae, aortic valve replacement, incisional and inguinal hernias including recurrent inguinal hernia, as well as mesh implantation, cholecystectomy, and nephropexy. During one procedure, due to intestinal obstruction, a 60 cm segment of small intestine had to be resected. At the present laparotomy, the patient had extensive adhesions with a complete fixation of the small intestine. Additional adhesions were encountered between the small intestine, the sigmoid colon, and a polytetrafluoroethylene (PTFE) mesh in the lower abdomen. The procedure lasted for 4 ½ hours, and serosal injuries were oversewn with 4-0 PDS. The abdomen was rinsed, and drainage was inserted. Before closure, 4DF gel (15 g powder plus 180 mL Ringer's solution) was applied onto the entire intestine and the peritoneal cavity. The early course was uneventful. Due to the massive extent of the adhesions and the complexity of the procedure, a second-look laparotomy was scheduled for the fifth postoperative day. The ventral portion of the abdomen was completely free of adhesions. Minor adhesions were detected in the dorsal compartment adjacent to the retroperitoneum. The small intestine appeared inconspicuous without indication of new adhesions (Figure 2). Sites of oversewn serosal injuries were covered with fibrin. After rinsing and renewed 4DF gel treatment, the abdomen was closed without insertion of drains. The further course of the patient was complicated by open wound treatment following seroma formation, anemia (intra-abdominal hemorrhage was excluded by repeated ultrasound evaluations, no evidence of intraluminal hemorrhage on endoscopy), catheter sepsis, fever, and intestinal atony. The patient was discharged on the 32nd postoperative day with completed wound healing and regular function of the gastrointestinal tract. Six months later, the patient underwent adhesiolysis without 4DF therapy at another hospital due to acute intestinal obstruction. Eight days later, she developed an ileus situation and showed severe inflammatory adhesions on relaparotomy. During this procedure, adhesions could not be completely dissected. The patient was on total parenteral nutrition for the following four weeks. Four months later, she was readmitted to our institution, again complaining of severe pain in the lower abdomen accompanied by signs of subileus. The diagnostic workup showed thromboembolic splenic infarcts, as well as a short length subtotal thromboembolic closure of the superior mesenteric artery. Treatment so far, 1 year and 8 months after the last operation, has been nonsurgical.

### 2.4. Patient 4

The fourth patient treated under acute conditions was an 82-year-old female with intestinal strangulation. The history showed two operations: a conventional cholecystectomy and, nine years ago, a left hemicolectomy and appendectomy for perforated diverticulitis. The strangulated segment of small bowel recovered after adhesiolysis and was vital. Extensive adhesiolysis had to be performed in all quadrants of the abdomen. Another jejunal conglomerate of 30 cm length with high vulnerability during dissection had to be resected and was anastomosed by end-to-end anastomosis. Several superficial injuries were oversewn with 4-0 PDS. Hemostasis and extensive rinsing of the abdominal cavity were performed, but no 4DF gel was applied during this initial procedure. A planned second-look operation was performed two days later. The loops of the small intestine showed adhesions and required adhesiolysis again. Serosal injuries were oversewn with 4-0 PDS. Vitality of the intestine was uncompromised, the anastomosis was intact and no third-look operation was planned. Before closure of the abdomen, 4DF gel (10 g powder mixed with 140 mL 0.9% saline solution) was applied onto the entire small bowel and the peritoneal cavity. The greater omentum was repositioned and treated with 4DF powder and subsequent transformation into a gel by dripping with saline solution. Robinson drains were inserted and removed on postoperative day 4. Following an uncomplicated course, the patient was discharged after 25 days and has since remained free of symptoms for 2 years and 1 month.

### 2.5. Patient 5

The fifth patient treated under acute conditions was 65 years old and presented with acute decompensation of chronic intestinal obstruction. She had had an anterior rectal resection for rectum carcinoma five years before and a history of several interventions following the resection, such as resection of lung metastases, endoscopic dilatation of the anastomosis, and several episodes of intermittent abdominal pain and constipation, which had been treated conservatively. The patient had received palliative chemotherapy for rib metastases for five months prior to the present surgery. In the present intervention, extensive adhesiolysis of a major portion of the small intestine had to be performed in order to restore the intestinal passage. Ten grams of 4DF powder was premixed as described before and applied as barrier gel onto the entire small intestine and the peritoneal cavity. The postoperative course was uneventful, and the patient was discharged from the hospital after 14 days. Palliative chemotherapy for metastatic disease was continued after completion of wound healing. Two months later, the patient presented with a subileus condition, which resolved under conservative therapy. The patient did not develop further episodes of intestinal obstruction but died nine months later due to metastatic disease.

## 3. Discussion

A common therapeutic option in chronic abdominal or pelvic pain caused by intestinal adhesions is operative adhesiolysis [27] despite recurrence of adhesions in 55–100% of patients [10]. Intestinal obstruction due to adhesions can be treated surgically or conservatively, and there is a long-lasting debate on which approach is superior [28–30]. Meier et al. [29] pointed out that the management of small bowel obstruction is based on biological tests, clinical evaluation, and computed tomography imaging and that the choice of treatment mainly depends on the surgeon's assessment. They found significantly lower recurrence of small bowel obstruction symptoms and new hospitalizations after surgical management than with conservative treatment [29]. Due to the drawbacks of both treatment options, an agent which can prevent recurrence of adhesions is highly desirable. As indicated before, adhesion barriers available on the market are discussed controversially regarding their efficiency [15], and most of them cannot be used in resective bowel surgery or in the case of incomplete hemostasis [12, 16–18]. Its adhesion barrier properties as gel and its hemostatic function as powder make 4DF an interesting choice for the prevention of adhesions after extensive intestinal adhesiolysis. Furthermore, there was no evidence for the presence of poor suture healing after application of 4DF in the present study.

In studies on 4DF published so far, the polysaccharide has been applied for adhesion prevention either as powder that was transformed into a gel in situ [19–24] or as rather viscous gel premixed extracorporeally with a mixing ratio of 20–40 mL 0.9% saline solution per 5 g 4DF powder [20, 21, 23, 24]. Due to the large areas at risk for adhesion formation in the abdomen, we prepared a gel with low viscosity by increasing the saline/powder ratio to 60–70 mL saline solution per 5 g 4DF powder. This facilitated homogeneous and convenient distribution of the 4DF gel on the intestinal loops and the peritoneal cavity. The gel was applied after extensive dissection of intestinal adhesions with chronic obstruction in three patients and acute intestinal obstruction with ileus in two patients.

Two (patients 1 and 2) of three patients treated for chronic obstruction have remained free of symptoms during a follow-up of more than 2 years. Patient 1 (Figure 1) deserved special attention since she was operated for symptomatic intestinal obstruction (pain and subileus) for the fourth time, including previous unsuccessful antiadhesive treatment with 4% icodextrin solution. Such a surgical history implicates a high risk for recurrent adhesions in short intervals [30]. All previous treatments had been followed by rapid recurrence of pain, whereas she now has remained free of symptoms for 2 years and 4 months.

Patient 3 showed no adhesions at second-look laparotomy five days after treatment with 4DF gel; only fibrin deposits on seroserosal sutures were found (Figure 2). However, adhesions recurred after several postoperative complications, which resulted in two additional operations. For the development of adhesions, impairment of the peritoneal layer and the formation of fibrin bridges are prerequisites [31]. The timing of the occurrence of adhesions in this case is not clear; since from a theoretical standpoint in peritoneal healing, the coverage with new mesothelium should be complete by day 5 irrespective of the size of the injury [32]. An intact mesothelium as basic prerequisite in order to prevent new adhesions should have been established on the day of the second-look operation, which did not show recurrence of adhesions (Figure 2) but fibrin deposits on the intestinal wall. It is conceivable that in contrast to the literature [32], due to the trauma of the extensive primary operation in combination with the complicated course and persisting fibrin deposits on the intestinal wall from seroserosal sutures, mesothelial healing may have been impaired. Furthermore, infection is a known trigger of adhesion formation [31]. Intra-abdominal bleeding as a cause of the postoperative anemia and possible cause for recurrent adhesions was ruled out by several ultrasound examinations, which did not detect free fluids. The anemia was attributed to catheter sepsis, prolonged infection, and fluid shifting from the third space after recuperation. In addition, the PTFE mesh in the lower abdomen may have contributed to new adhesions. In total, two of the five patients had second-look laparotomies. In patient 3, the second-look laparotomy, a limited operation of short duration five days after the first intervention, was followed by series of complications. In comparison, patient 4 with a second-look laparotomy two days after the first intervention recovered from both operations with an uneventful course. Therefore, in conditions, in which a second-look operation is necessary, the number of 4DF gel applications (i.e., at second-look only or at both operations) as well as the timing and tolerance of the repeated laparotomy are at issue. Patient 5 was readmitted two months after an uneventful primary course due to subileus, which was treated by nonsurgical therapy. A circumscribed pathology of the intestine on ultrasound examination, such as segmental dilatation as a sign of intestinal obstruction, could not be established. In addition to new adhesions, mucosa affections due to chemotherapy or strictures of the anastomoses could have caused the rapidly resolving symptoms.

## 4. Conclusion

A total of three out of four patients with a follow-up of more than 2 years have remained free of symptoms after extensive intestinal adhesiolysis in combination with application of 4DF gel as adhesion barrier. All patients showed severe conditions of adhesions with challenging intraoperative situations. The agent can generally be applied in acute and chronic intestinal obstruction; healing of anastomoses does not seem to be impaired. These favorable preliminary results with 4DF as low-viscosity barrier gel justify further evaluation in larger prospective randomized trials.
